# Revisiting AGAMOUS-LIKE15, a Key Somatic Embryogenesis Regulator, Using Next Generation Sequencing Analysis in *Arabidopsis*

**DOI:** 10.3390/ijms232315082

**Published:** 2022-12-01

**Authors:** Sanjay Joshi, Hadia Awan, Priyanka Paul, Ran Tian, Sharyn E. Perry

**Affiliations:** 1 Kentucky Tobacco Research and Development Center (KTRDC), University of Kentucky, Lexington, KY 40546, USA; 2 Department of Plant and Soil Sciences, University of Kentucky, Lexington, KY 40546, USA; 3 Biopathogenix, Nicholasville, KY 40356, USA; 4 Department of Plant and Soil Science, Institute for Genomics of Crop Abiotic Stress Tolerance, Texas Tech University, Lubbock, TX 79430, USA

**Keywords:** next generation sequencing, microarray, MADS-domain protein, somatic embryo

## Abstract

AGAMOUS-like 15 (AGL15) is a member of the MADS-domain transcription factor (TF) family. MADS proteins are named for a conserved domain that was originally from an acronym derived from genes expressed in a variety of eukaryotes (**M**CM1-**A**GAMOUS-**D**EFICIENS-**S**ERUM RESPONSE FACTOR). In plants, this family has expanded greatly, with more than one-hundred members generally found in dicots, and the proteins encoded by these genes have often been associated with developmental identity. *AGL15* transcript and protein accumulate primarily in embryos and has been found to promote an important process called plant regeneration via somatic embryogenesis (SE). To understand how this TF performs this function, we have previously used microarray technologies to assess direct and indirect responsive targets of this TF. We have now revisited this question using next generation sequencing (NGS) to both characterize in vivo binding sites for AGL15 as well as response to the accumulation of AGL15. We compared these data to the prior microarray results to evaluate the different platforms. The new NGS data brought to light an interaction with brassinosteroid (BR) hormone signaling that was “missed” in prior Gene Ontology analysis from the microarray studies.

## 1. Introduction

Somatic embryogenesis (SE) is a biotechnological tool that is utilized for basic research as a means to regenerate transgenic plants to test gene function with the aim to understand plant development and responses to the environment. SE also provides a valuable resource for plant improvement and the propagation of commercial crops [1]. SE is a complex process that involves inducing somatic cells to undergo reprogramming to potentially regenerate an entire normal plant by first forming an embryo structure called a somatic embryo (also called SE).

*AGAMOUS-LIKE15* (*AGL15*) encodes a member of the MIKC (for **M**ADS-**I**ntervening linker-**K**eratin-like-**C**arboxyl-terminal) subfamily of MADS domain transcription factors (TFs) that promotes somatic embryo development when constitutively expressed in Arabidopsis (*Arabidopsis thaliana*), soybean (*Glycine max*), and cotton (*Gossypium hirsuitum*), while mutants (loss-of-function) of both *AGL15* and *AGL18* (*AGL18* encodes a protein closely related to AGL15) significantly reduces somatic embryo development in Arabidopsis [2,3,4]. One of the SE systems in Arabidopsis is embryo culture tissue (ECT), where when placed in culture, immature zygotic embryo explants produce and maintain SE without exogenous hormones for extended periods of time in the presence of constitutive *AGL15* expression [2] (currently more than 27 years to date). A *35S:AGL15* transgene also promotes somatic embryo development from the shoot apical meristem (SAM), in a culture system called SAM somatic embryogenesis (SAM SE) from mature seeds that complete germination in liquid media containing 2,4-dichlorophenoxyacetic acid (2,4-D) [2,3].

Prior work in the lab performed chromatin immunoprecipitation (ChIP) followed by genomic tiling microarray hybridization (ChIP-Chip) for AGL15 embryo culture tissue (ECT) to identify potential binding sites genome-wide. In addition, microarrays to assess transcript accumulation were used to assess changes in response to AGL15 accumulation [5]. Combining these data allowed for the determination of direct responsive targets compared to potentially indirect targets (responsive but not directly associated with AGL15). This type of analysis for transcription factors (TFs) is an important step to understanding how a TF potentiates its phenotype or response to the environment. However, the array approaches have limitations in terms of the features represented, and which portion of the transcripts may be assessed. Additionally, these studies are limited to species for which arrays have been developed.

In this study, we employed the next-generation sequencing (NGS) to obtain a better resolution of genome-wide binding by AGL15 using ChIP followed by sequencing (ChIP-Seq), and transcript accumulation in response to AGL15 using RNA-sequencing (RNA-Seq) techniques. High throughput sequencing is becoming more ubiquitous as the cost of high-throughput sequencing technology continues to drop. NGS also does not have the same limitations as the arrays. In fact, the arrays used in Zheng et al. (2009) [5] are no longer commercially available. NGS has many advantages compared to microarrays. RNA-Seq identifies all transcripts, whereas microarrays cover only a subset of the transcriptome. A specific example from our studies in Arabidopsis showed that the array lacked features to measure the transcript accumulation from microRNA (miRNA) encoding genes, so while we could assess the binding to regions near the annotated genes, we had to infer the potential response from the response of targets of the miRNA. RNA-Seq is not limited in this manner and can identify splice variants and previously unidentified transcripts. This provides the leverage to identify unique transcripts that were not covered in previous studies. Furthermore, in this study, we combined RNA-Seq and ChIP-Seq data to identify the direct induced and repressed targets for AGL15. We compared the results from ChIP-Chip and ChIP-Seq, and microarray and RNA-Seq, and provide categories of targets that have not been previously reported.

## 2. Results

### 2.1. Genome-Wide Mapping of Regions bound by AGL15 Using Chip-Seq

Embryo culture tissue (ECT) was used to map in the vivo AGL15 binding sites using the ChIP-Chip approach [5]. Overexpression of the *AGL15* transgene supports the stable establishment of ECT [2], and these cultures have also been used for the characterization of genome-wide binding sites for several other studies including FUSCA3 [6], ABSCISIC ACID INSENSITIVE3 [1], and AGL18 [7]. Here, we used the ECT tissue to map AGL15 sites, but by using the next-generation sequence (NGS) technique ChIP-Seq. Prior work on mapping of AGL15 ChIP-Seq has been reported in [7], which was analyzed using CLC Genomics Workbench 12 (ChIP-Seq Analysis). Here, we used the AGL15 ChIP-Seq raw data to analyze using another platform called CisGenome [8], and compared the ChIP-Chip and ChIP-Seq data using the same platform (CisGenome). Three biological replicates of the ChIP-Seq experiment were analyzed together using CisGenome (this report), whereas two out of three rules were applied to the analysis of these data in the CLC Genomics Workbench that analyzes the replicates one-by-one [7]. In CisGenome, all three replicates and controls were considered together, allowing for more robust statistical analysis.

AGL15 binds to thousands of regions in the Arabidopsis genome. In our study, we found 6596 potential peaks that were common among the three replications with a false discovery rate (FDR) <0.01. A comprehensive list is provided in Supplementary Dataset S1. These peaks were assigned to 5454 genes (some genes had more than one binding site associated). When the list of genes with potential regulatory regions associated was analyzed using the Gene Ontology (GO) term enrichment tool by Protein Analysis Through Evolutionary Relationships (PANTHER17.0; [9]), many categories were overrepresented including the “regulation of seed development” (GO:0080050), fold enrichment (FE)—3.63, false discovery rate (FDR) 6.15 × 10^−3^. Other selected categories within “biological processes” are shown in Table 1.

**Figure 1:**
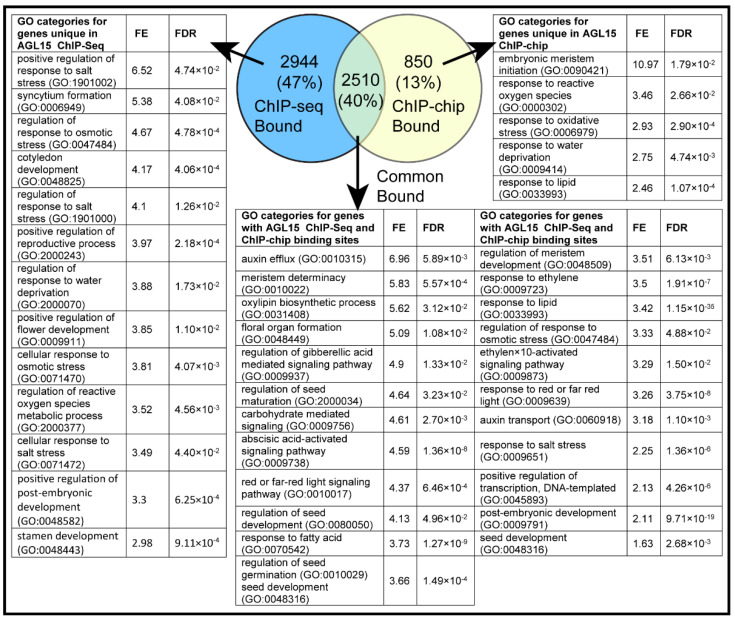
GO enrichment analysis for genes with regulatory regions associated with AGL15 ChIP-chip, ChIP-Seq, or both approaches. Panther classification system was used to find the significantly overrepresented categories. Fold enrichment compares the dataset to the whole *Arabidopsis* genome (release 16 November 2021). The number of genes identified as associated with AGL15 in each and common to both approaches is shown. Please note that the number of binding sites is larger for each approach than the number of genes associated because some genes have more than one AGL15 binding region identified. FE indicates fold enrichment (please see the text).

CisGenome was previously used with ChIP-Chip to identify 3708 peaks (FDR < 0.01) assigned to genes associated with AGL15 binding sites that were assigned to 3360 genes [5]. When we compared both ChIP datasets, 40% of the genes identified in ChIP-Seq were also found in ChIP-Chip (Figure 1). Genes associated with the AGL15 binding sites identified with both techniques numbered 2510, and when we generated the GO term list for the genes associated in both methods, many categories were overrepresented including the “regulation of gibberellic acid-mediated signaling pathway” (GO:0009937); FE 4.9, FDR—1.33 × 10^−2^; and “response to fatty acid” (GO:0070542), FE 3.73, FDR—1.27 × 10^−9^. Other overrepresented categories within “biological responses” are shown in Figure 1. There were 2944 unique genes in the ChIP-Seq study that were not reported in the ChIP-Chip experiment. Overrepresentation of thee GO term “biological processes” for the unique genes from the ChIP-Seq showed categories mostly involved in stress, specifically salt stress genes. In addition, many genes involved in reproductive development were present (Figure 1). A comprehensive list of unique genes from both studies is given in Supplementary Dataset S2.

**Figure 2:**
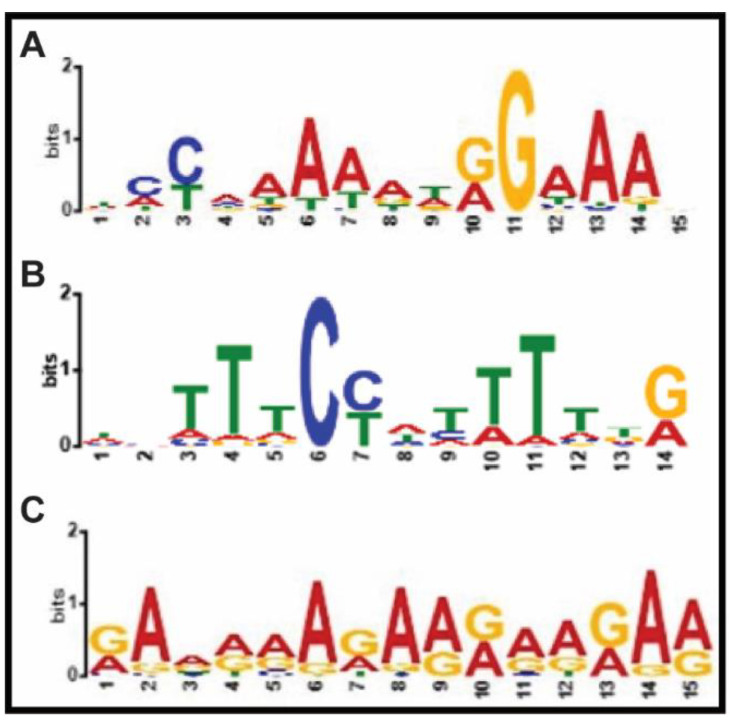
ChIP-MEME analysis for binding regions: (**A**) common in ChIP-Seq and ChIP-Chip; (**B**) unique in ChIP-Seq; and (**C**), unique in ChIP-Chip. The sequences of the genome fragments were retrieved using CisGenome [8] and ChIP-MEME performed using the default settings [10].

Furthermore, we were interested in identifying the cis motifs for common genes in ChIP-Chip and ChIP-Seq as well as in unique genes from both studies. Paul et al. (2021) [7] demonstrated an overrepresentation of CArG motif binding sites for MADS domain proteins with a canonical sequence of CC(A/T)6GG using the CLC Genomics Workbench for thee AGL15 ChIP-Seq data. Here, we took the binding sequence data and analyzed them using the MEME-Suite 5.3.3 and default settings [10]. As shown in Figure 2*,* cis motif enrichment for common binding regions from ChIP-Chip and ChIP-Seq show an overrepresentation of CArG motifs (Figure 2A). Moreover, the unique regions of ChIP-Seq showed also CArG motifs, however, unique regions of the ChIP-Chip genes did not show any specific overrepresentation of the CArG motifs (Figure 2B,C). The types of CArG motifs illustrated in Figure 2A,B are preferentially bound in vitro (electrophoretic mobility assay—EMSA analysis) by AGL15 [11].

### 2.2. Gene Expression Changes in Response to AGL15 Accumulation

Only knowing the binding regions of AGL15 does not demonstrate a regulatory consequence of AGL15 association, so it is important to determine the genes that respond to changes in AGL15 accumulation in some tissues (e.g., SAMSE). Overexpression of *AGL15 (35S:AGL15)* results in a significant increase in somatic embryogenesis in both the ECT and SAM SE systems, while loss-of-function as a double mutant with the closely related *AGL18* gene (*agl15/agl18*) results in a significant decrease in SE in both systems [3]. From immunolocalization studies within the embryo context, AGL15 does not accumulate excessively, even within a *35S:AGL15* transgene [12]. In addition, AGL15 accumulates in very young seedling SAMs but is reduced in older SAM [2,3]. As such, we believe that enhanced SE is more from the stable accumulation of AGL15 over a developmental sequence rather than excessive ectopic accumulation. Micro-array technology was previously used to determine the gene expression for SAM SE tissue [5]. In this study, RNA-Seq was used to assess the transcriptome in response to AGL15 accumulation in Columbia wild type Col, (wt), the *35S:AGL15* line, and the *agl15/agl18* double mutant. The double mutant *agl15/agl18* SAM SE expression data have been described in Paul et al. (2021) [7]. Here, we highlight the *35S:AGL15* line compared to Col (wt). Two biological replicates were performed for 10-day old SAM SE tissues for each genotype and the percentage of total mapped with genes for each replicate was determined. The results showed that 1294 genes showed increased transcript levels, and 1664 genes showed decreased transcript levels in response to the transgene (*p* < 0.05, cutoff 1.5 fold change). The data are presented in Supplementary Dataset S3.

Furthermore, we compared the RNA-Seq and microarray gene expression studies [5], which used more stringent parameters in the published microarray study. We have since generated data with the same cutoffs as conducted for the RNA-Seq (1.5-fold cutoff instead of 2 in [5]) to allow for a comparison in this study. We created the gene list comparison of transcript levels from the RNA-Seq study with relevance to both or either experiment (*35S:AGL15* or *agl15/agl18*) from the microarray [5] compared to Columbia (wt). This broader comparison provided 3295 genes in common with the microarray experiment (Supplementary Dataset S4). We conducted a GO term analysis for these gene lists (induced and repressed) and found several processes related to SE. For example, the repressed transcripts were enriched for meristem development, response to hormones such as auxin, ethylene, gibberellin, and abscisic acid. Similarly, induced genes showed GO term enrichment for various processes such as water transport and response to oxygen levels (Table 2).

### 2.3. Identification of Putative Direct and Indirect Targets of AGL15 from NGS Data

**Figure 3:**
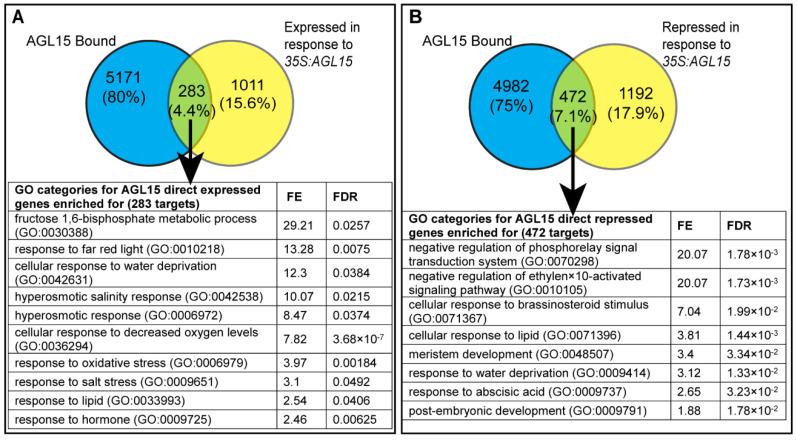
Overrepresented GO categories for genes associated with AGL15 by ChIP-Seq and responsive to AGL15 accumulation in 10 days after culture (dac) *35S:AGL15* SAM SE tissue compared to the wild type. (**A**) Direct induced and (**B**) direct repressed in response to AGL15. Details of the analysis are the same as in Figure 1. FE is fold enrichment compared to the whole Arabidopsis genome.

Prior studies combined ChIP-Chip and microarray data to provide the genes that are bound as well as responsive (either induced or repressed in response to AGL15 accumulation). Here, we combined the ChIP-Seq and RNA-Seq (*35S:AGL15* compared to Col, wt) data to obtain better insights on the direct induced/repressed targets as well as indirect targets that are responsive but not bound. As shown in Figure 3, two hundred and eighty-three (4.4%) genes were directly induced and 472 (7.1%) were directly repressed targets. When the lists of the direct and responsive targets were examined for the overrepresentation of GO terms, directly induced genes were overrepresented in the hyperosmotic salinity response (GO: 0042538), and directly repressed genes were overrepresented in the negative regulation of the ethylene-activated signaling pathway (GO: 0010105) (Figure 3) (Supplementary Dataset S5).

GO analysis of the directly repressed targets found by NGS highlighted genes involved in response to brassinosteroid (BR) signaling was overrepresented (Figure 2B). This was a new overrepresented category that was not found by prior microarray technologies and provided an additional new focus to investigations into understanding how AGL15 regulates SE. A recent publication reported on the direct interactions between AGL15, BR signaling, and SE, demonstrating that BR promotes the embryo to seedling transition and the mechanism of action is at least partially via *AGL15* expression [13]. Genes encoding two positive regulators of BR signaling, *BRI1-EMS-SUPPRESSOR 1* (*BES1*), and *BRASSINAZOLE-RESISTANT 1* (*BZR1*) were found to bind directly to *AGL15′s* promoter region, and the regulation of *AGL15* by these BR TFs in controlling SE is implicated by the fact that loss-of-function of *agl15* restores normal seedling development to mutants in the BR pathway as opposed to SE development in the *AGL15* background.

**Figure 4:**
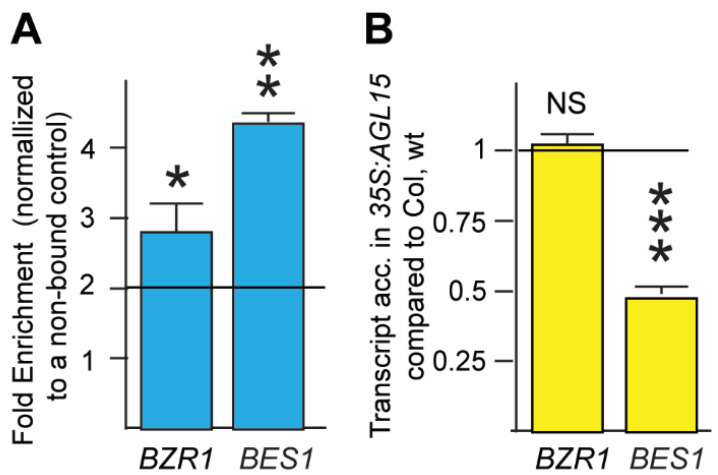
Confirmation of binding (**A**) and response (**B**) to AGL15. (**A**), qPCR results on two to three independent ChIP populations to assess the association of AGL15 with DNA regions associated with potential 5′ regulatory regions of *BZR1* and *BES1* compared to a region that is not bound by AGL15 (an intergenic region that is not bound based on ChIP-Chip and ChIP-Seq studies). Values greater than 2 (line) are considered as significant binding. (**B**) Response of transcript accumulation to AGL15. Overexpressing tissue of *AGL15* (*35S:AGL15*) was compared to the wild type (Col, wt); biological replicate number of at least three biological replicates for both binding and response. * significant at *p* < 0.05; ** significant at *p* < 0.01; *** significant at *p* < 0.001, NS not significant at *p* < 0.05).

The new ChIP-Seq and RNA-Seq data to determine genes directly repressed by AGL15 (Figure 3B) revealed that genes encoding proteins involved in response to BR were overrepresented. Indeed, both *BZR1 (At1g75080)* and *BES1 (At1g19350)* had 5′ upstream regions directly associated with AGL15, both in the ChIP-Chip and ChIP-Seq studies (the overlap is shown in Supplementary Figure S1; both the CisGenome analysis of all three replicates and the CLC analysis of the individual replicates are shown). It should be noted that CisGenome assigns a binding site to the nearest gene. Thus, these binding sites were assigned to the 3′ ends of the flanking genes. The CLC Genomics Workbench flags both the 5′ and 3′ flanking genes. We confirmed the association of these regions with AGL15 by ChIP-qPCR on independently generated ChIP populations (Figure 4A).

While the RNA-Seq data did not show a significant response of these genes to AGL15 accumulation, the microarray data [5] indicated that *BZR1* was significantly upregulated in *agl15 agl18* compared to Col, wt (1.5-fold and significant for the comparison of *agl15 agl18* to Col, wt; whereas the comparison of *35S:AGL15* to Col, wt was 0.8, but not significant—*BES1* was not present). Because of the impact of these genes on SE, we followed up using qRT-PCR on the 10 d SAM SE tissue. As shown in Figure 4B, while *BZR1* transcript accumulation did not respond to the *35S:AGL15* transgene, *BES1* transcript was significantly reduced in this background, indicating that AGL15 can repress *BES1* expression. It should be noted that these two genes are highly similar (84% identity) and required use of the 3′ UTR to design specific primers.

## 3. Discussion

### 3.1. Comparison of ChIP-Chip and ChIP-Seq

We determined and compared the genome-wide binding sites of the MADS-domain transcription factor AGL15 using ChIP-Seq and ChIP-Chip. Although the sequencing technology is a different approach and new biological replicates than those used in the ChIP-Chip study were generated, we found a high correlation between the datasets. About 75% of the ChIP-Chip genes associated with the AGL15 binding sites were also present in the ChIP-Seq data using CisGenome to analyze the data and considering all three replicates together [8]. The ChIP data demonstrated that AGL15 binds to thousands of regions in the Arabidopsis genome. In a recent study [7], about 87% of genes associated with AGL15 binding sites identified previously using a ChIP-Chip approach were also identified as potential direct targets in ChIP-Seq. This study used the CLC Workbench platform and only required a majority rule (present in two out of three replications).

Similar studies comparing ChIP-Chip and ChIP-Seq were performed for another MADS-domain transcription factor SEP3 [14,15]. Kaufmann et al. showed good agreement between the results of the two methods and showed a large overlap in the peak positions and a similar ranking of the peaks (90% of the peaks identified in ChIP-Chip had a ChIP-Seq peak within 1000 bp). They also highlighted that the ChIP-Seq approach provides a better resolution of the binding pattern, coverage, and sensitivity. Cutoffs for the ChIP-Chip peak size were larger. In the case of ChIP-Seq, the average peak size was around 800 base pair (bp) compared to the ChIP-Chip average peak size of approximately 1300 bp, which resulted in a lower resolution for multiple binding sites [14]. In the case of the AGL15 ChIP-Chip study, the average peak size was 552 (bp), whereas for ChIP-Seq, it was 286 bp. The principle to generate genome-wide DNA-binding profiles for ChIP-Seq and ChIP-Chip are different. ChIP-Seq utilizes rapidly developing platforms for massively parallel DNA sequencing whereas ChIP-Chip uses printed arrays. ChIP-Seq has better resolution (depending on size of fragments), genome coverage with higher sensitivity, and improved signal-to-noise ratio over ChIP-Chip [15,16,17]. The increased genome coverage is due to the fact that repetitive regions may be masked on the arrays and that there are gaps between the features represented on the arrays that can affect hybridization (for review, [15]).

While the shared binding sites between ChIP-Seq and ChIP-Chip and those unique in ChIP-Seq showed overrepresented motifs that corresponded to potential cis-regulatory elements recognized by AGL15, the ChIP-Chip unique binding sites did not identify this element (Figure 2). This could be due to the larger average peak size in ChIP-Chip, “diluting” the actual binding site. The entire dataset for ChIP-Chip did, in fact, show an enrichment of potential MADS binding sites [5].

### 3.2. Newly Discovered AGL15 Direct Targets Using NGS

As described above, the different approaches to characterize genome-wide binding sites have different limitations. Both the unique ChIP-Seq targets and the ChIP-Seq/ChIP-Chip shared targets found an enrichment of genes involved in stress response, in particular, the response to osmotic stress (GO:0047484) and the regulation of response to salt stress (GO:1901000) (ChIP-Seq) or just response to salt stress (GO:0009651) (ChIP-Chip). The ChIP-Chip unique sites do not show enrichment in these categories, but do highlight involvement in response to other stresses (Figure 1). ChIP-Seq unique targets also highlight the potential involvement of AGL15 in the positive regulation of reproductive process (GO:2000243) (Figure 1). These data provide a better understanding of genomic regions bound by AGL15, providing further insight into mechanisms by which this factor promotes SE. Stress has been recognized as a triggering factor for somatic embryogenesis for a long time (see [18] for review).

We then determined the targets that respond to AGL15 accumulation using NGS compared to the microarray to assess transcriptomes. RNA-Seq has advantages over expression arrays because expression arrays are limited by the genes represented and do not (often) include genes such as small RNA encoding genes. In addition, only identified genes are represented. By combining ChIP-Seq and RNA-Seq, we determined direct responsive targets. We found a minority of bound sites responded to AGL15 as direct induced (4.4%) and repressed (7.1%) genes, however, a larger number of responsive genes were identified compared to the prior study [5]. In agreement with the prior study, a larger number of genes are directly repressed by AGL15 than directly induced, suggesting that while AGL15 has dual function, it may act mainly as a repressor. In support of this observation, while no obvious transcriptional activation domain is present in AGL15, a motif associated with gene repression is present in the carboxyl-domain of this protein [19]. However, recent studies indicate that while this motif is involved in the repression of some targets, a mutant form of AGL15 lacking this motif can still repress some direct targets, while other targets are no longer responsive [20]. This may reflect different compositions of the MADS–domain interactions and/or different mechanisms of repression.

Our results show that the minority of genes with which AGL15 is directly associated show response, as measured by transcript accumulation. Several studies had similar comparable fractions as direct targets that are responsive to the accumulation of the transcription factor. For instance, for HY5, only 5.6% of the bound sites showed a response at the cutoffs used [21]. Association without obvious regulation appears to be a trend and may reflect situations where under other contexts, perhaps when associated with other factors, there would be a response (reviewed in [22]). 

When we performed the analysis of the GO term “biological responses” using the unique ChIP-Seq bound genes (2944) and direct induced targets (283), we found a gene list involved in the cellular response to stress (GO:0033554), which is one of the key inducing factors for SE. Some of the genes found were *AT1G22220 (AUXIN UP-REGULATED F-BOX PROTEIN 2-AUF2)* [23] and AT4G02380 *(SENESCENCE-ASSOCIATED GENE 21-LEA5/SAG21* [24] (Supplementary Table S1). Furthermore, when we examined the genes for direct repressed (472) and unique ChIP-Seq binding targets with the GO term for biological processes, we found GO term enrichment involved in meristem development (GO:0048507) and cellular lipid metabolic process (GO:0044255). We extracted the genes involved in these GO terms, which are listed in Supplementary Tables S2 and S3, respectively. Overall, we identified several key pieces of information that could potentially have an impact on understanding the SE mechanism. For example, one of the direct repressed targets of AGL15 is *ARGONAUTE10 (AGO10)*, which was shown to be involved in regeneration processes and the maintenance of stem cell populations in Arabidopsis by controlling the miR165/166 pathway [25,26].

### 3.3. AGL15 and Hormones

AGL15 and hormones play a crucial role in promoting SE. Hormones such as auxin, ethylene, and gibberellic acid (GA), and their interaction with AGL15 in SE have been reviewed in Joshi et al. (2022) [27]. The role of ethylene is crucial depending on the context. Prior studies have shown that AGL15 impacts ethylene biosynthesis and signaling, which affects SE [28]. When GO term analysis for (direct induced and repressed) targets from AGL15 ChIP-Seq and *35S:AGL15* RNA-Seq data was performed, interestingly, direct repressed targets showed a “negative regulation of ethylene-activated signaling pathway” (GO:0010105) (Figure 3), and the induced targets showed a “response to hormone” (GO:0009725). While prior work showed a positive impact of ethylene on promoting SE, we also found that too high of an accumulation actually (as provided by addition of a precursor of ethylene) inhibited this process [28,29]. This fits the theme that there are subtle balances in biology with much cross-talk to maintain homeostasis, which is easy through too little or too much to disrupt the processes. The gene identifiers and name for genes encoding proteins involved in these processes are listed in Supplementary Tables S4 and S5.

One new GO annotation that was discovered in the NGS work, but not the array approach, was “cellular response to brassinosteroid stimulus” (GO:0071367), which was significantly overrepresented in the AGL15 direct, repressed list (Figure 3). Recent studies have shown the role of BR in SE that involves AGL15 [13], which combines with our results (Figure 4), indicating a feedback between BR and AGL15, possibly to promote the transition between the embryo and seedling. Furthermore, *BZR1* and *BES1*, discussed above, we found two additional BR-related genes *AT2G01950, SERINE/THREONINE-PROTEIN KINASE BRI1-LIKE 2* (*BRL2*—direct AGL15 repressed target)*,* and *AT2G13790, SOMATIC EMBRYOGENESIS RECEPTOR KINASE 4* (*SERK4*—also a direct AGL15 repressed target) from this study, which might have potential roles in BR signaling and SE. *BRL2* encodes a leucine-rich repeat receptor-like kinase (LRR-kinase) that is similar to BRI, a brassinosteroid receptor [30]. SERK4 is also a receptor-like kinase involved in BR signaling [31]. A loss of function allele of *SERK4* is associated with embryo lethality when combined with mutants in *serk1/serk2/serk3(bak1)* [31]. Both genes showed little to no transcript accumulation in the developing seeds, with more transcript present post-germination, based on data summarized in the eFP browser [32,33], looking at the seed/plant development datasets “Klepikova Atlas” [34], “Developmental Map” ([35], with data contributed by the Nambara lab), and “Seed” [36,37]. This pattern of transcript accumulation is consistent with repression by AGL15; AGL15 (both transcript and protein) accumulates to its highest level in developing embryos [38,39,40].

## 4. Materials and Methods

### 4.1. Plant Material

*Arabidopsis thaliana* wild-type (wt), and *35S:AGL15* plants (all Col ecotype) were used for SAM SE. The seeds were allowed to develop to dry seed and SAM SE performed as described in [2]. For RNA-Seq, tissue was collected 10 days after the start of culture (dac) and flash frozen. For ChIP-Seq, we used embryo culture tissue (ECT), which was generated as described in prior work [5]. Briefly, ECT cultures were cross-linked and stabilized (protein–protein, protein–DNA interactions) using 1% formaldehyde (Sigma-Aldrich, St. Louis, MO, USA). 

Nuclei were isolated using extraction buffer [5] and sonication was performed to fragment chromatin to ~500 base pair fragments. We used anti-AGL15 antibody and protein A-Sepharose beads (Thermo-Fisher, Waltham, MA, USA) to precipitate AGL15. The control used was the same tissue without any antibody.

### 4.2. ChIP-Seq, Data Analysis, and qPCR

ChIP was performed as previously described [5]. To analyze the ChIP-Seq data, CisGenome was used. This platform was previously used for the ChIP-Chip study [5]. In the prior study, a perfect match–mismatch was used to compute the probe intensity, and peak detection was performed using a moving average cutoff of 2.5 and a minimum region of 250 bp. CisGenome was used to analyze all three biological replicates of the ChIP-Seq data for AGL15. The default settings for ChIP-Seq were used that amounted to a read extension of 150 bp, maximum gap of 50 bp, and a minimum peak size of 100 bp with at least a cutoff of greater than three comparing the IP (immuno-precipitate) to the control samples. ChIP-Seq data were previously analyzed with CLC Genomics Workbench 12 (Qiagen, Germantown, MD, USA), which allowed each replicate to be assessed individually [7]. To confirm the association of AGL15 with select DNA fragments, independent ChIP populations were generated and qPCR was performed as in [5], but using a CFX Connect Real-Time PCR system (Bio-Rad. Hercules, CA, USA) instead of an I-cycler. The oligonucleotide primers are provided in Supplementary Table S6.

### 4.3. RNA-Isolation, RNA-Seq, and qRT-PCR

For 10 d old SAM SE seedlings, RNA isolation and RNA-Seq were performed as follows. RNA was isolated using the QIAGEN RNeasy Plant Mini Kit following the manufacturer’s instructions, but supplementing the RLC buffer with 1% final (*w*/*v*) high MW (15,000–20,000) PEG (polyethylene glycol; Sigma-Aldrich, St. Louis, MO, USA). Two biological replicates were prepared for wild type and *35S:AGL15* and sent for library preparation and RNA-Seq (Novogene, Sacramento, CA, USA, https://www.novogene.com/us-en/). Data were analyzed by using CLC Genomics Workbench 12. To cast the broadest net, we used *p* > 0.05 and fold change of at least 1.5-fold cutoffs. The double mutant *agl15/18* SAM SE expression data have been described in Paul et al. (2021) [7]. GO term analysis was performed using PANTHER [9]. The confirmation of accumulation of the transcript in response to AGL15 for select targets was performed by generating independent populations of RNA, converting to cDNA, and performing qRT-PCR as described in Wang and Perry (2013) [6]. The oligonucleotide primers are provided in Supplementary Table S6. 

### 4.4. Data Accessions

Sequence data for ChIP-Seq, and RNA-Seq (Col, wt and *agl15 agl18*) can be found in the GenBank/EMBL data libraries under the Bio project accession number PRJNA777254. The data for microarray and ChIP-Chip is available through GEO series accession number GSE17742 [5]. The RNA-Seq for *35S:AGL15* SAM SE is available through accession number PRJNA903892.

## Figures and Tables

**Table 1:** GO categories for genes with AGL15 ChIP-Seq binding sites. The GO identification number is in parentheses. This GO analysis includes all 5454 genes associated with AGL15 as found by ChIP-Seq. Fold enrichment indicates how overrepresented genes the genes are in a category compared to the whole genome. FDR; false discovery rate.

GO Categories	Fold Enrichment	FDR
Regulation of seed maturation (GO:2000034)	4.09	2.74 × 10^−3^
Regulation of abscisic acid biosynthetic process (GO:0010115)	3.77	4.58 × 10^−2^
Regulation of response to salt stress (GO:1901000)	3.66	9.51 × 10^−4^
Regulation of seed development (GO:0080050)	3.63	6.15 × 10^−3^
Very long-chain fatty acid biosynthetic process (GO:0042761)	3.4	4.59 × 10^−2^
Positive regulation of seed germination (GO:0010030)	3.28	3.65 × 10^−2^
Response to gibberellin (GO:0009739)	3.11	1.45 × 10^−7^
Regulation of response to water deprivation (GO:2000070)	3.06	9.09 × 10^−3^
Regulation of seed germination (GO:0010029)	2.86	5.50 × 10^−5^
Regulation of hormone levels (GO:0010817)	2.63	8.56 × 10^−12^
Response to hormone (GO:0009725)	2.5	4.51 × 10^−50^
Regulation of post-embryonic development (GO:0048580)	2.39	3.46 × 10^−12^
Seed development (GO:0048316)	1.67	5.08 × 10^−7^

**Table 2:** GO enrichment analysis for the transcripts common in RNA-Seq and microarray studies that are upregulated and downregulated compared to Col, wt. The PANTHER classification system was used to discover significantly (FDR < 0.05) overrepresented categories. Fold enrichment compared the dataset to the whole Arabidopsis genome (release 16 November 2021).

GO Categories for Induced Genes Enriched for:	Fold Enrichment	FDR
Regulation of cytokinin-activated signaling pathway (0080036)	6.94	4.44 × 10^−2^
Cellular response to oxygen levels (0071453)	5.62	2.12 × 10^−22^
Water transport (0006833)	5.55	1.09 × 10^−2^
Fatty acid beta-oxidation (0006635)	4.5	2.78 × 10^−2^
Positive regulation of abscisic acid signaling (0009789)	4.32	6.52 × 10^−3^
Response to gibberellin (0009739)	3.25	3.37 × 10^−3^
Positive regulation of response to stimulus (0048584)	2.29	6.60 × 10^−3^
**GO categories for repressed genes enriched for:**	**Fold Enrichment**	**FDR**
Embryonic meristem initiation (0090421) Ethylene biosynthetic process (0009693)	7.40 5.14	2.16 × 10^−4^ 1.85 × 10^−2^
Auxin polar transport (0009926)	3.43	2.19 × 10^−3^
Response to fatty acid (0070542)	2.84	1.91 × 10^−5^
Response to lipid (0033993)	2.42	1.02 × 10^−15^
Response to gibberellin (0009739)	2.33	4.75 × 10^−2^
Response to abscisic acid (0009737)	2.33	3.63 × 10^−8^
Response to water deprivation (0009414)	2.14	6.35 × 10^−5^
Response to salt stress (0009651)	2.14	5.35 × 10^−6^

## Data Availability

Not applicable.

## Supplementary Materials

The supporting information can be downloaded at: https://www.mdpi.com/article/10.3390/ijms232315082/s1.
