# Supplementary material for: Revisiting AGAMOUS-LIKE15, a Key Somatic Embryogenesis Regulator, Using Next Generation Sequencing Analysis in Arabidopsis
Source: Int J Mol Sci. 2022 Dec 1;23(23):15082. doi: 10.3390/ijms232315082 (PMC9736886; doi:10.3390/ijms232315082)
Supplement: Supplementary file 1 [file ijms-23-15082-s001.zip › Supplementary Figure S1.pdf]

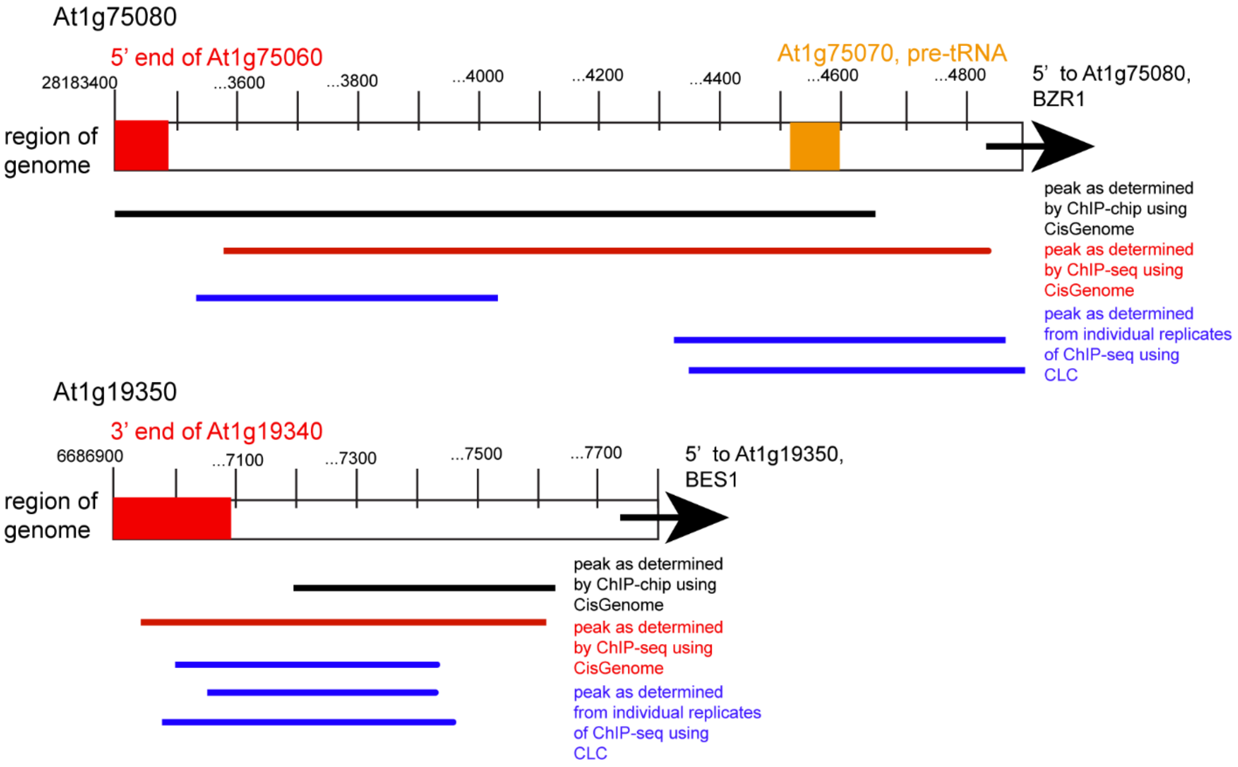

Supplementary Figure S1. Overlap of binding sites identified for AGL15 using different approaches.
Black; region identified as bound by AGL15 in ChIP-chip as in Zheng et al, (2009) using CisGenome. Red; region identified as bound by AGL15 in ChIP-seq considering all three biological replicates in CisGenome. Blue; regions of the genome identified as associated with AGL15 in the indiviidual replicates of the experiment using CLC Workbench to look at the data.
