# Supplementary material for: Revisiting AGAMOUS-LIKE15, a Key Somatic Embryogenesis Regulator, Using Next Generation Sequencing Analysis in Arabidopsis
Source: Int J Mol Sci. 2022 Dec 1;23(23):15082. doi: 10.3390/ijms232315082 (PMC9736886; doi:10.3390/ijms232315082)
Supplement: Supplementary file 1 [file ijms-23-15082-s001.zip › Supplementary Tables S1-S6.pdf]

Supplementary Table S1. Gene list involved in "cellular response to stress (GO:0033554)"- Direct expressed targets of AGL15 previously not reported.

**Gene ID**	**Gene Name**
AT2G26660	*SPX DOMAIN-CONTAINING PROTEIN 2;SPX2*
AT1G76650	*CALCIUM-BINDING PROTEIN CML38;CML38*
AT5G58070	*TEMPERATURE-INDUCED LIPOCALIN-1;TIL*
AT1G78380	*GLUTATHIONE S-TRANSFERASE U19;GSTU19*
AT5G42200	*E3 UBIQUITIN-PROTEIN LIGASE ATL23;ATL23*
AT1G05680	*UDP-GLYCOSYLTRANSFERASE 74E2;UGT74E2*
AT4G02380	*SENESCENCE-ASSOCIATED GENE 21;SAG21*
AT4G33030	*UDP-SULFOQUINOVOSE SYNTHASE;SQD1*
AT2G47730	*GLUTATHIONE S-TRANSFERASE F8,GSTF8*
AT3G60520	*T8B10_180*
AT2G18670	*RING-H2 FINGER PROTEIN ATL56;ATL56*
AT5G64260	*EXORDIUM-LIKE 2;EXL2*
AT5G20150	*SPX DOMAIN-CONTAINING PROTEIN 1;SPX1*
AT5G20830	*SUCROSE SYNTHASE 1;SUS1*
AT1G22220	*F-BOX PROTEIN AT1G22220*
AT1G63090	*F-BOX PROTEIN PP2-A11;PP2A11*
AT4G37930	*SERINE HYDROXYMETHYLTRANSFERASE 1,SHM1*
AT5G12020	*17.6 KDA CLASS II HEAT SHOCK PROTEIN;HSP17.6*

Supplementary Table S2. Gene list involved in "meristem development (GO:0048507)"- Direct repressed targets of AGL15 previously not reported.

**Gene ID**	**Gene Name**
AT2G46920	*PROTEIN PHOSPHATASE 2C 32;POL*
AT1G36160	*ACETYL-COA CARBOXYLASE 1;ACC1*
AT5G45780	*PROBABLE LRR RECEPTOR-LIKE SERINE/THREONINE-PROTEIN KINASE*
AT3G08850	*REGULATORY-ASSOCIATED PROTEIN OF TOR 1;RAPTOR1*
AT5G46700	*PROTEIN TORNADO 2;TRN2*
AT5G03150	*ZINC FINGER PROTEIN JACKDAW;JKD*
AT4G32880	*HOMEOBOX-LEUCINE ZIPPER PROTEIN ATHB-8;ATHB-8*

AT5G43810	*PROTEIN ARGONAUTE 10;AGO10*
AT2G35350	*PROTEIN PHOSPHATASE 2C 29;PLL1*
AT3G06020	*PROTEIN FANTASTIC FOUR 4;FAF4*

Supplementary Table S3. Gene list involved in "cellular lipid metabolic process (GO:0044255)"-Direct repressed targets of AGL15 previously not reported.

**Gene ID**	**Gene Name**
AT1G36160	*ACETYL-COA CARBOXYLASE 1;ACC1*
AT2G37940	*PHOSPHATIDYLINOSITOL:CERAMIDE INOSITOLPHOSPHOTRANSFERASE 2;IPCS2*
AT5G43760	*3-KETOACYL-COA SYNTHASE 20;KCS20*
AT1G06080	*DELTA-9 ACYL-LIPID DESATURASE 1;ADS1*
AT5G25900	*ENT-KAURENE OXIDASE, CHLOROPLASTIC;KO*
AT5G41080	*GLYCEROPHOSPHODIESTER PHOSPHODIESTERASE GDPD2;GDPD2*
AT3G63240	*TYPE I INOSITOL POLYPHOSPHATE 5-PHOSPHATASE 4;IP5P4*
AT2G42010	*PHOSPHOLIPASE D BETA 1;PLDBETA1*
AT3G48460	*GDSL ESTERASE/LIPASE*
AT2G45970	*CYTOCHROME P450 86A8;CYP86A8*
AT2G28630	*3-KETOACYL-COA SYNTHASE 12;KCS12*
AT3G14205	*PHOSPHOINOSITIDE PHOSPHATASE SAC2;SAC2*
AT1G54570	*ACYLTRANSFERASE-LIKE PROTEIN*
AT1G47990	*GIBBERELLIN 2-BETA-DIOXYGENASE 4;GA2OX4*

Supplementary Table S4. Gene list of AGL15 direct repressed targets involved in "negative regulation of ethylene-activated signaling pathway" (GO:0010105).

**Gene ID**	**Gene Name**
AT3G51770	*ETHYLENE-OVERPRODUCTION PROTEIN 1;ETO1*
AT2G40940	*ETHYLENE RESPONSE SENSOR 1;ERS1*
AT5G03730	*SERINE/THREONINE-PROTEIN KINASE CTR1;CTR1*
AT2G25490	*EIN3-BINDING F-BOX PROTEIN 1;EBF1*
AT3G04580	*PROTEIN EIN4;EIN4*
AT4G02680	*ETO1-LIKE PROTEIN 1;EOL1*

AT1G58250	*PROTEIN SABRE;SAB*

Supplementary Table S5. Gene list of AGL15 direct expressed targets involved in "response to hormone" (GO:0009725).

**Gene ID**	**Gene Name**
AT2G37640	*EXPANSIN-A3;EXPA3*
AT2G33590	*NAD(P)-BINDING ROSSMANN-FOLD SUPERFAMILY PROTEIN;CRL1*
AT3G17510	*CBL-INTERACTING SERINE/THREONINE-PROTEIN KINASE 1;CIPK1*
AT4G37760	*SQUALENE EPOXIDASE 3;SQE3*
AT3G04730	*AUXIN-RESPONSIVE PROTEIN IAA16;IAA16*
AT4G38970	*FRUCTOSE-BISPHOSPHATE ALDOLASE 2, CHLOROPLASTIC;FBA2*
AT4G36800	*NEDD8-CONJUGATING ENZYME UBC12;RCE1*
AT4G30270	*XYLOGLUCAN ENDOTRANSGLUCOSYLASE/HYDROLASE PROTEIN 24;XTH24*
AT1G10370	*GLUTATHIONE S-TRANSFERASE U17;GSTU17*
AT2G05100	*CHLOROPHYLL A-B BINDING PROTEIN 2.1, CHLOROPLASTIC;LHCB2.1*
AT4G32940	*VACUOLAR-PROCESSING ENZYME GAMMA-ISOZYME;AT4G32940*
AT2G37220	*RNA-BINDING PROTEIN CP29B, CHLOROPLASTIC;CP29B*
AT1G14920	*DELLA PROTEIN GAI;GAI*
AT5G01810	*CBL-INTERACTING SERINE/THREONINE-PROTEIN KINASE 15;CIPK15*
AT3G57870	*SUMO-CONJUGATING ENZYME SCE1;SCE1*
AT1G58340	*PROTEIN DETOXIFICATION 48;DTX48*
AT5G53160	*ABSCISIC ACID RECEPTOR PYL8;PYL8*
AT5G67480	*BTB/POZ AND TAZ DOMAIN-CONTAINING PROTEIN 4;BT4*
AT1G10210	*MITOGEN-ACTIVATED PROTEIN KINASE 1;MPK1*
AT1G05680	*UDP-GLYCOSYLTRANSFERASE 74E2;UGT74E2*
AT5G13790	*AGAMOUS-LIKE MADS-BOX PROTEIN AGL15;AGL15*
AT4G19700	*E3 UBIQUITIN-PROTEIN LIGASE BOI;BOI*

AT5G50760	*AT5G50760;MFB16.16*
AT4G02380	*SENESCENCE-ASSOCIATED GENE 21, MITOCHONDRIAL;SAG21*
AT2G47460	*TRANSCRIPTION FACTOR MYB12;MYB12*
AT1G51950	*AUXIN-RESPONSIVE PROTEIN IAA18;IAA18*
AT5G17300	*PROTEIN REVEILLE 1;RVE1*

Supplementary Table S6. Oligonucleotides used in this study.

AGI	Gene name	Forward	Reverse
For ChIP-qPCR:			
At1g75080	*BZR1*	CTTGTCTTGTCTTGGTTGTGAAG	CCAAACGTATCCACTCCTTTCT
At1g19350	*BES1*	GGGCAAATCCACTATCCAGTT	GTTGGGACCTGCTCTTCATT
Intergenic region: Identified as non-bound in ChIP	*Intergenic*	GAACTACTCGGTTTGCGAATTG	CCTTGCCGATCCTGATGAATA
For qRT-PCR:			
At1g75080	*BZR1*	CTGGGAAGGAGAGAGGATACA	CATTTGGGTTTGCCTAGTTGTT
At1g19350	*BES1*	CGGAAGGAAGGATTGGAGAATG	CGTTGTGAAACAAACTCACTGG
AT5G19770	*TUA3*	TGGTGCCCAACTGGGTTCAAAT	ACCTCTGCAACTGCTGTGTTGT
